# “You Can See the Connections”: Facilitating Visualization of Care Priorities in People Living with Multiple Chronic Health Conditions

**DOI:** 10.1145/3544548.3580908

**Published:** 2023-04-19

**Authors:** Hyeyoung Ryu, Andrew B. L. Berry, Catherine Y. Lim, Andrea L. Hartzler, Tad Hirsch, Juanita I. Trejo, Zoë A. Bermet, Brandi Crawford-Gallagher, Vi Tran, Dawn Ferguson, David J. Cronkite, Brooks Tiffany, John Weeks, James D. Ralston

**Affiliations:** University of Washington, Seattle, WA, USA; Northwestern University, Chicago, IL, USA; Kaiser Permanente Washington, Health Research Institute, Seattle, WA, USA; University of Washington, Seattle, WA, USA; Northeastern University, Boston, MA, USA; Kaiser Permanente Washington, Health Research Institute, Seattle, WA, USA; Kaiser Permanente Washington, Health Research Institute, Seattle, WA, USA; Kaiser Permanente Washington, Health Research Institute, Seattle, WA, USA; Kaiser Permanente Washington, Health Research Institute, Seattle, WA, USA; Kaiser Permanente Washington, Health Research Institute, Seattle, WA, USA; Kaiser Permanente Washington, Health Research Institute, Seattle, WA, USA; Kaiser Permanente Washington, Health Research Institute, Seattle, WA, USA; Kaiser Permanente Washington, Health Research Institute, Seattle, WA, USA; Kaiser Permanente Washington, Health Research Institute, Seattle, WA, USA

**Keywords:** multiple chronic health conditions, reflection, sensemaking, patient-clinician communication, visualization, patient priorities care, values

## Abstract

Individuals with multiple chronic health conditions (MCC) often face an overwhelming set of self-management work, resulting in a need to set care priorities. Yet, much self-management work is invisible to healthcare providers. This study aimed to understand how to support the development and sharing of connections between personal values and self-management tasks through the facilitated use of an interactive visualization system: Conversation Canvas. We conducted a field study with 13 participants with MCC, 3 caregivers, and 7 primary care providers in Washington State. Analysis of interviews with MCC participants showed that developing visualizations of connections between personal values, self-management tasks, and health conditions helped individuals make sense of connections relevant to their health and wellbeing, recognize a road map of central issues and their impacts, feel respected and understood, share priorities with providers, and support value-aligned changes. These findings demonstrated potential for the guided process and visualization to support priorities-aligned care.

## 1 INTRODUCTION

Many individuals with multiple chronic health conditions (MCC) manage their health at home with the everyday demands of chronic illness care [8, 67] including: illness work (e.g., physical activity, taking medications, monitoring blood glucose levels and blood pressure), everyday life work (e.g. obligations to family, job), and biographical work (e.g., coping with the emotions around accepting and adjusting to changes in life due to illness) [26, 56]. The combination of self-care tasks for individuals with MCC can be overwhelming leading individuals with MCC to prioritize certain self-care tasks over others [86]. Much of this daily work and prioritization by patients is not visible to healthcare providers.

Improving the healthcare for individuals with MCC requires orienting care toward outcomes important to patients. Recent efforts to improve outcomes have sought to align healthcare with an individual patient’s values [61] or what an individual considers most important for well-being and health [12, 55]. Eliciting and sharing an individual patient’s values, however, is not a routine part of healthcare [11, 14, 52]. The inadequate involvement of an individual’s values in care may contribute to the common discordance in priorities for care between people with MCC and their healthcare providers [15, 42, 75, 86].

Designers have an opportunity to help individuals with MCC identify and communicate the complex relationships among their values, self-care activities, and health conditions. Based on development work with individuals with MCC and healthcare providers, we designed an intervention combining a conversation and a visualization tool supported by an interactive information system called Conversation Canvas (CC). The visualization tool, CC, helped individuals with MCC articulate, reflect on, and share these complex relationships. In this field study piloting the intervention in a clinical setting, we sought to understand the roles of visualization and facilitated conversations by human interventionists trained as social workers (hereafter, “interventionists”) in the activities of reflection by the individuals with MCC and sharing with their healthcare providers.

The field study took place in Washington State and included 13 participants including 3 who opted to include a family caregiver during the evaluation interviews. Intervention activities included eliciting individual patient’s values, self-management tasks and health conditions; developing and reflecting on the connections between these domains; and sharing these reflections in both written and visual form with primary care providers in preparation for upcoming visits. In this study, we aimed to investigate how the guided use of our visualization tool used with interventionists could support reflection on these connections by individuals with MCC and communicate these reflections to healthcare providers. This paper makes three primary contributions:

1. We showed how using the interactive visualization tool with trained human interventionists in two sequential interventions expanded the sensemaking and reflection process of individuals with MCC.
2. We illustrated how the guided process made individuals with MCC feel more empowered in their care, and supported priorities-aligned conversations with their providers in clinical visits.
3. We uncovered how the interactive visualization tool and the facilitated conversations respectively and conjointly supported individuals with MCC.

## 2 RELATED WORKS

### 2.1 Patient-Provider Communication About Personal Values and Care Priorities

Clinical guidelines suggest that healthcare providers take a patient-centered approach (e.g., collaborative care model) to help individuals with MCC make choices that better align self-management with their values [12]. The collaborative care model encourages the collaboration of patients and their healthcare team members in managing chronic illness as health care for chronic conditions requires not only ongoing self-management work [13, 14, 20], but coordination amongst the multiple agents involved (e.g., informal caregivers, primary care physicians, medical assistants, registered nurses) [82]. This approach requires patients to meet with their providers to not only assess their health but to adjust their care plan as needed. To plan care with the patient, the patient and provider discuss and define health-related problems collaboratively. For instance, providers may ask about the positive or negative outcomes of the prior care plan to discuss new directions of care. Patients may also share their concerns regarding new symptoms or negative changes in their lives due to illness management [82]. After the patient and provider define a problem collaboratively, they target specific problems to focus on. However, as patients deal with multiple chronic conditions, the number of collaboratively defined problems rises, resulting in an often-overwhelming set of self-care tasks for patients to manage. Good care in this setting requires healthcare providers and patients to prioritize problems.

Patients with MCC and providers, however, often do not agree on priorities for health care (e.g., which health conditions and treatment options to prioritize). Patients tend to prioritize symptomatic conditions over asymptomatic ones [36, 87] and their personal values influence their priorities for health care [15, 52, 54]. Although personal values are pivotal to understanding patients’ priorities, many self-care technologies inherit the medical perspective, which aims to efficiently quantify, track, and keep the conditions under control [7, 77]. Such overemphasis on medical aspects can neglect people’s everyday experience with conditions [7, 77]. Patients also have complex lives outside the clinical setting where they play different roles (e.g., mother, son, caregiver) that may conflict with their role as a patient [30, 39]. Although HCI research on self-care has made important contributions to supporting effective and efficient self-care, it has under-addressed the complexity, diversity, and conflicts that come from the differing motivations, activities, and routines in the lives of individuals outside of the clinical setting [7, 40, 76]. This is especially evident when the complexity, diversity and conflicts intersect with the ever-present work of self-managing multiple ongoing health conditions [7, 40, 76]. Thus, Fitzpatrick [30] and Nunes [64] suggest changing the design of self-care technologies by incorporating a holistic understanding of individuals’ everyday lives, including an understanding of their social contexts, how they divide care with others, how they adapt to health conditions, what resources they use, and how their perspectives differ from one another.

The importance of Fitzpatrick [30] and Nunes’ [64] approach is echoed in studies showing that patients with MCC find it hard to articulate or share their priorities with their healthcare providers [9, 17, 53, 75]. People with MCC may perceive boundaries regarding which values they can or should discuss because they may not know how their values relate to their health care and may not see how health care can support their values [53]. Providers and patients with MCC also often face conflicting recommendations for managing different health conditions [9, 17]. When patient and provider priorities do not align, patients are more likely to experience worse health outcomes [75]. Therefore, in this study, we aimed to help patients better articulate and share their priorities with healthcare providers through CC designed to reflect a holistic understanding of patients’ everyday lives.

### 2.2 Focusing on Personal Values to Improve Health and Wellbeing of Patients with MCC

Patients’ personal values influence their priorities for health care. We define personal values as what is most important to the well-being and health of patients and align the definition with the definition of values from value-sensitive design rather than medical literature. In medical literature, values are often perceived to be “preferences” [29], which contrasts, for example, with the perspective of value-sensitive design where values are what a person considers important in life [33]. We acknowledge that more nuanced definitions of values have been discussed and developed in prior HCI research [15, 32, 44, 49]. Designers have defined values from moral and ethical discourses [32, 49] or from local contexts and lived experiences [49]. Also, values have not been regarded as fixed and stable entities, but as living entities that are constantly put into practice and “repaired” over time [44]. In our work, we align the definition of personal values with perspectives that (1) consider values to be personal and/or rooted in local contexts [15, 49] and (2) allow values to change and develop through enactment and re-enactment in practice [15, 44].

Prior work on eliciting patient’s values in clinical settings have set a narrow definition for personal values. Previous research typically provided pre-set options for patients to consider [84] which did not account for values to change over time [50]. Also, they did not provide patients with the opportunity to iteratively express their values as they developed in practice [15, 84]. Recent work has sought to broaden the range of personal values to encompass values that are important to people with MCC but have not been incorporated significantly into clinical contexts [12, 55]. Lim et al. [54] uncovered six domains of personal values that patients with MCC deemed as most pertinent to their well-being and health – principles (e.g., ideals, spirituality), relationships (e.g., family, friends), emotions (e.g., comfort, joy), activities (e.g., reading, exercising), abilities (e.g., vision, mobility), and possessions (e.g., garden, automobile). In this study, we build on this work to explore the role of interactive visualization for reflecting on the connections between patients’ personal values, self-care tasks and health conditions.

Clinicians may better align their care plans with patients’ priorities when they understand how patients connect personal values to self-care activities [26]. The recently developed Patient Priorities Care (PPC) framework is a promising approach to better align clinical decision with the priorities of patients with MCC. PPC begins with a structured process in which the individuals with MCC works with a healthcare team member to identify their most desired outcome goals based on their priorities (i.e., values, goals, and preferences). In a nonrandomized trial, PPC reduced treatment burden, fewer self-management tasks, and fewer medications [80]. Challenges in implementing PPC included difficulty translating a patient’s priorities into clinical decisions [16]. The PPC framework also elicits patient values based on the framework developed among patients with cancer and where values were seen as fixed and stable over time [62]. Such elicitation does not adequately reflect shifts in values of individuals with MCC over time, including the need to identify new and meaningful roles as disease and aging progress. Understanding how to better support patients reflecting on values and their connections to self-care task and health conditions could fill an important gap in emerging patient-centered care models for individuals living with MCC.

However, eliciting personal values is not often part of routine clinical practice [6, 22, 48]. Although typically providers ask their patients what they would like to be included the agenda for the clinical visit [47], patients filter what they disclose to providers because they perceive personal values as not pertinent to healthcare [52]. Time constraints also limit what patients disclose to clinicians [38]. The PPC feasibility evaluation also noted limitations in its disregarding the time constraints typical in primary care and the patients’ barriers in communicating personal values with their providers [16]. Clinicians were concerned about patients having to come back for another visit and using the short routine visit time to implement the PPC framework. Providing thorough reflection on values, self-management and health tasks before a provider visit may be essential for enhancing the quality of care and the long-term feasibility of implementing care aligned with the values of individuals with MCC.

### 2.3 Designing Support for Collaborative Reflection and Sharing

Healthcare technologies are enabling new forms of communication between patients and providers [18]. Patient portals and personal health records have enabled synchronous, remote communication [51, 79]. There are also technologies geared toward connecting patients’ experiences of illness with the medical perspective of providers [1, 2, 23, 66]. However, most healthcare information technologies remain disease-focused rather than supporting awareness of what matters most to patients with MCC [37, 38, 57]. Notable work aiming to support patients’ long-term self-management of Human Immunodeficiency Virus addressed gaps in holistic monitoring and patient engagement in self-tracking technologies [24]. Claisse et al. [24] claimed that self-tracking technologies need to support not only physical but also psychosocial aspects of health. For holistic engagement, self-tracking technologies also need to support collaborative reflection with providers on what is most important to the patient [24, 65]. Nunes et al. [65] highlighted the overemphasis on medical aspects of chronic conditions which treats users of self-care technologies as patients who are expected to prioritize treatment and self-management over their interests and everyday lives [65]. To design better tools for patients, Nunes et al. suggested designers need to better understand and incorporate the reality of patients as people we meet in everyday life that “have different interests, hobbies, values, and roles, and that, by chance, happen to have a chronic condition that sometimes requires time and concern”[65].

O’Kane et al. [68] elucidated the need for more efficient tools to holistically monitor health in people managing chronic conditions, such as migraine and diabetes. Individuals with chronic conditions felt overwhelmed with the task of collecting, combining, and making sense of the large, varied sets of information (both personal and generic) to understand normality within their condition and personal illness experiences [68]. As self-care technologies often underestimate the complexity of patients’ lives outside of clinical settings and overgeneralize the experiences of individuals with MCC, O’Kane et al. [68] and Nunes et al. [65] suggest incorporating end-user customization and individuality into the self-care technology designs. For engagement in holistic health, Mentis et al. [59] provided insights on how patients and clinicians could “craft a view” of care management in consultation with patients’ self-tracked data. They suggested the inclusion of features to point and annotate used by both patients and providers. They further emphasized the need for patients’ personal views to not be heavily influenced by clinicians’ crafted views, and the need for clinician and patient to collaboratively craft a view together.

Currently, the design suggestions for holistic monitoring of and engagement in patients’ health have not been well-incorporated into the tools available for eliciting patients’ goals and preferences for care. Most solutions were developed in the context of individuals with advanced illnesses for end-of-life planning [3, 10, 78]. These individuals usually manage critical life-threatening illnesses or advanced stages of major chronic diseases. Advanced care planning for these individuals elicits preferences for end-of-life care which would otherwise be made by their surrogate decision makers. These tools, though, do not directly address the daily self-management decisions and priorities of individuals with MCC who are weighing the benefits, burdens, and tradeoffs associated with long-term management of several chronic conditions. Future healthcare technologies need to address the gap where systems could elicit and promote conversation about patients’ values and their connections to self-management priorities for individuals with chronic conditions.

Recent work has argued that promoting reflection is important for individuals with MCC to gain awareness and articulate their values [19, 54]. There is a need to understand how visualization guided by interventionists may help reflect on and share complex relationships between values, self-care tasks, and health conditions. A notable previous study tested interactive visualization prototypes to investigate how to support individuals with MCC’s reflection on these complex relationships [15]. The previous study was a single reflection, done in a research environment with research staff. This field study extends the prior work by deploying and testing a functional system in a clinical setting with guidance from interventionists engaging individuals in two sequential reflection visits. We explore how the guided and interactive visualization used in this care setting supports collaborative reflection and communication of priorities to healthcare providers.

## 3 FOUNDATIONS OF THE DESIGN

Design of the visualization tool and facilitation was grounded in prior work on patient reflection and in the frameworks of self-determination theory (SDT) [73] and collaborative care for chronic illness [83]. SDT posits that an “autonomy supportive” environment is essential for establishing care partnerships that lead to patients’ autonomous motivation for healthful self-management [73] and it aligns with the collaborative care approach. Collaborative care, based on behavioral principles and evidence on effective chronic illness care, strengthens and supports self-care while ensuring effective medical, preventive, and health maintenance interventions [83]. The final design of the visualization tool and facilitation was informed by Berry et al.’s [15] study of three paper prototypes for visually exploring and articulating connections among values, self-care tasks, and health conditions in people with MCC. Design of these prototypes was informed by prior work on eliciting patient values and their connections to self-care tasks and healthcare conditions [12–15, 54, 55]. These prototypes tested three dimensions of reflection: self-directed reflection compared to reflection facilitated by a trusted interventionist; open-ended compared to constrained reflection; reflection in different time frames from past to into the future [15]. We used the following four guidelines (listed with direct quotes from prior work [15]) identified in testing these three prototypes to inform the design of the collaborative tool and facilitation:

1. “Begin with exploratory, reflective conversation between the patient and an active, empathetic listener” (p.26).
2. “Map the conversation as it progresses. Externalize key topics (i.e., personal values, self-care duties, health status indicators) and visualize relationships among them” (p.26).
3. “Conclude with a step to identify and articulate takeaways. In the context of reflection on values and health for people with MCC, these takeaways can be a list of topics to discuss with the doctor at an upcoming visit” (p.26).
4. “Align this concluding step with familiar and established practices such as making a list of topics to discuss with the doctor at an upcoming clinical encounter” (p.26).

Berry et al.’s findings highlighted the need for a trained interventionist in helping individuals with MCC use the tool to develop and visualize connections between values, self-care tasks, and health conditions in preparation for sharing with healthcare providers [15]. Consistent with SDT [73], this facilitated conversation combined with visualizing connections is likely to enable individuals with MCC in self-management activities [15] and care planning with healthcare providers. Sharing the results of the facilitated conversation with healthcare providers is meant to support collaborative problem identification, a first critical step in chronic illness care planning. Having individuals with MCC see and share what matters to them should enable defining of problems with providers and shared understanding, thus reducing discordance between the priorities of individuals with MCC, family caregivers, and healthcare team members.

## 4 METHODS

We conducted a field study that used qualitative interviews to evaluate the intervention using our prototype, CC. Study procedures received approval from the Kaiser Permanente Washington Health Research Institute. In this section, we report methods in four parts: (1) participants and recruitment, (2) intervention, (3) evaluation, and (4) analysis.

### 4.1 Participants and Recruitment

We engaged 13 individuals with MCC (P1-P13), with a mean age of 62.31 (SD=11.07) years. The study setting was two Kaiser Permanente Washington (KPWA) primary care clinics and 7 primary care providers (PCP) of participants with MCC participants. We identified potential participants with MCC using Electronic Health Records (EHR) and administrative data. To be eligible, individuals with MCC had to be over the age of 21, have five or more encounters with primary care team members in the past year including at least 3 PCP encounters, and have a diabetes diagnosis and at least two of the following chronic conditions: depression, osteoarthritis, and coronary artery disease. We selected these conditions because they are likely to require daily self-management to achieve optimal health outcomes and self-management demands for these conditions may either overlap or conflict with one another. For potential participants identified initially through automated data, we provided each PCP with a list of potentially eligible patients on their panel. We asked PCPs to let us know which patients would not be able to engage in a conversation with us, were not under their care, or would otherwise not be appropriate for the study. Study staff then called potential eligible participants to further assess eligibility and obtain consent. We limited recruitment to those who were English-speaking, age 21 or over, living at home without the help of a professional caregiver, and had five or more encounters with primary care team members in the past year including at least 3 PCP encounters. We also verified that the participants had reliable internet access and the ability to use a web browser. We excluded individuals with dementia. We informed participants that they were free to leave the study at any time without penalty and that their PCPs were not informed whether they decided to participate. Table 1 shows the demographics of our participant sample.

Three of the participants with MCC elected for the optional inclusion of a family caregiver. All caregivers (n=3) (CG5, CG9, CG13) co-habited with the corresponding participants with MCC and were their spouses. Participants with MCC received $50 after completing the baseline survey and elicitation of values, self-care tasks, and health condition, and received $100 after completing the follow-up interview at the end of the study. Caregiver participants received $50 after completing the follow-up interview at the end of the study, as part of the evaluation of the clinical setting implementation.

### 4.2 Intervention

**Figure 1:**
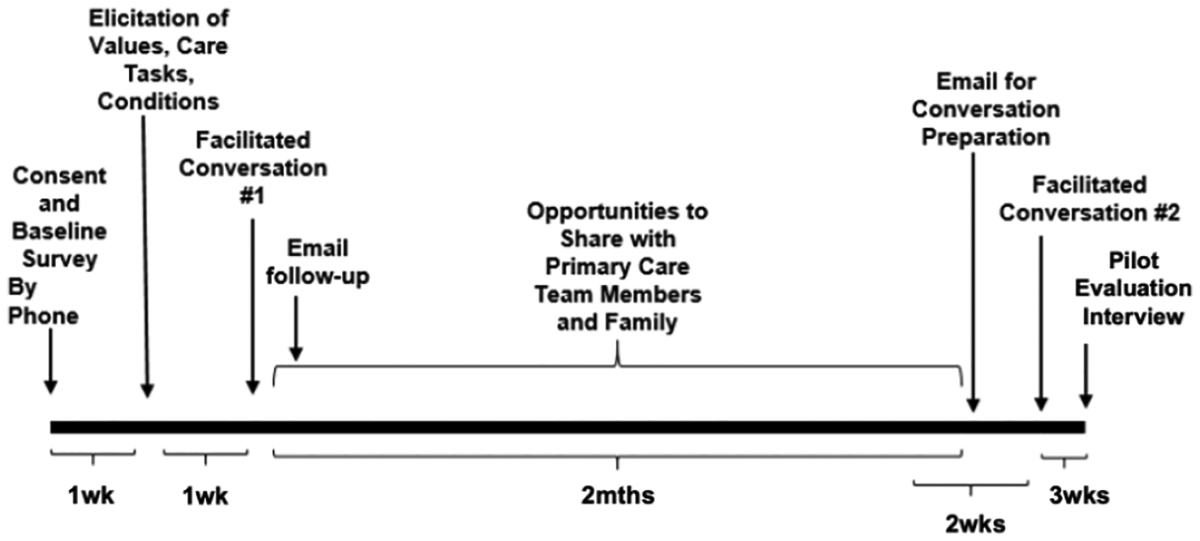
Individual with MCC Participant’s Intervention Process. After consenting to the study, participants engaged in value elicitation followed by the 1st facilitated conversation. Participants then had opportunities to share the visualization and summary of the conversation with family and primary care team members during upcoming primary care visits., Participants engaged in the 2nd facilitated conversation after the primary care visit or 2 months whichever came first. Lastly, they completed the evaluation interview.

In this section, we illustrate each individual with MCC participant’s journey through the intervention (Figure 1): value elicitation, 1st and 2nd facilitated conversations, sharing with healthcare team, healthcare team visit, and the evaluation interview. CC is the visualization tool supported by an interactive information system helping individuals with MCC articulate, reflect on, and share the connections between personal values, self-management tasks, and health conditions. An interventionist first elicits participant’s personal values in a telephone interview conducted in preparation of the facilitated conversations. The interventionist and participant then have two facilitated conversations (interchangeable with intervention calls) over the telephone while using CC.

#### 4.2.1 Value Elicitation.

Two interventionists with training in motivational interviewing [60] conducted intervention activities. Motivational interviewing was chosen for its focus on exploring and resolving ambivalence. The study team first mailed a worksheet to the participants to help them reflect on values, self-management tasks and health conditions in advance of the telephone interview. Then, the interventionist conducted a telephone interview (approximately 30 to 45 minutes) prior to the participants’ upcoming primary care visit. The process of elicitation was informed by prior work on establishing and validating the probes for personal values [12, 55] and follow-up design and prototype testing [15]. During the value elicitation, the interventionist used a series of conversational probes to elicit participants’ values in three areas – (1) what is most important to the participants’ wellbeing and health or personal values across six domains previously identified (principle, relationships, emotions, activities, abilities, and possessions [55]), (2) their self-management activities with probes of the domains, and (3) the health conditions they actively manage.

For value elicitation, we used practices established in prior work that include: probes for specific value domains; freedom for participant responses to be included in multiple value domains; open-ended probes to fill in values potentially not covered in the probes for the domains; and a summary and reflection step on values guided by the interventionist [12, 15]. We used a similar approach for eliciting self-management work with probes for the domains of illness, everyday life, and biographical work [27]. To ensure the elicitation of health conditions remains focused on participant perspectives and experiences, we used only open-ended questions to collect the health conditions participants managed and did not prompt participants using preexisting lists of conditions from the EHR that can be inaccurate [15]. After elicitation, the interventionist documented these items in the CC in preparation for a future conversation.

#### 4.2.2 Facilitated Use of the Conversation Canvas.

**Figure 2:**
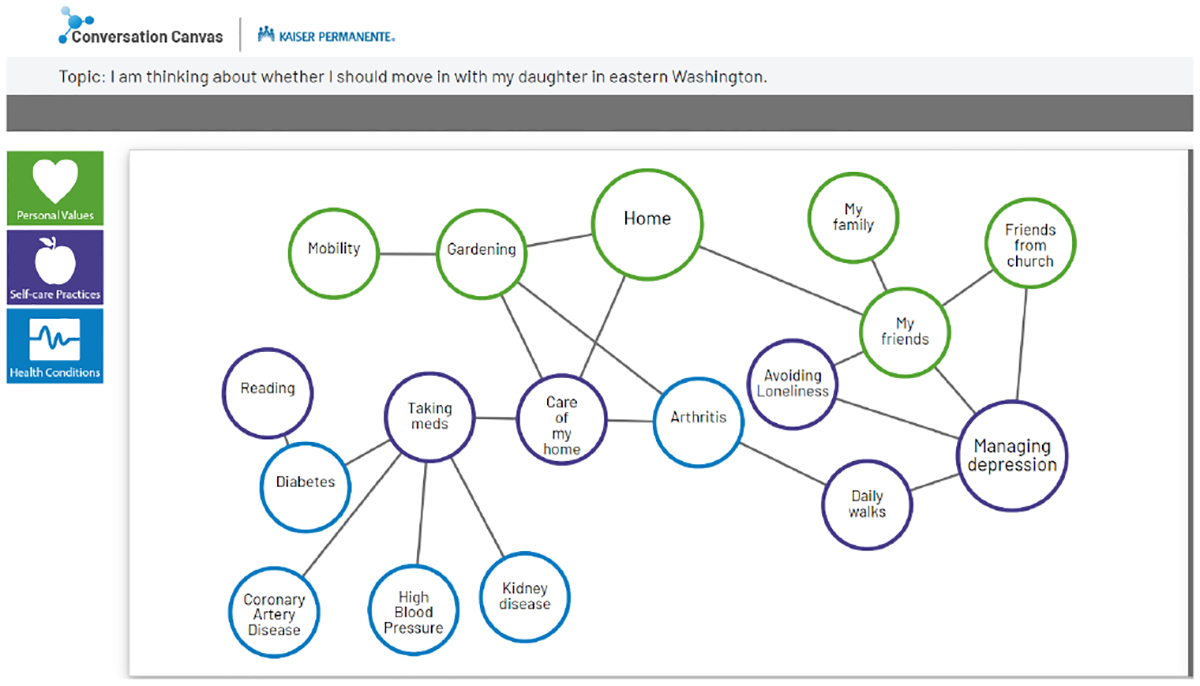
Conversation Canvas. CC is a website developed for mapping and exploring the connections between the individuals with MCC’s personal values (displayed in green circle nodes), self-management tasks (displayed in purple circle nodes), and health conditions (displayed in blue circle nodes). The question in the top middle is the conversation topic the participant chose to discuss. The participants and interventionists were able to add the relevant nodes and connect them by drawing connections through edges (grey connecting lines) during the 1st and 2nd facilitated conversations. Text boxes on the right are for written reflections on the connections.

The CC is a website developed for mapping and exploring the connections between the individuals with MCC’s personal values (displayed in green circle nodes), self-management tasks (displayed in orange circle nodes), and health conditions (displayed in blue circle nodes) (Figure 2). The participant and/or interventionist was able to add the relevant nodes and connect them by drawing connections through edges (grey connecting lines) during the 1st and 2nd facilitated conversations. The user could make the sizes of the nodes bigger or smaller and move them around on the canvas to place them in the places they deemed fit. The CC was used in the facilitated conversations with the interventionist, but the participants were also able to make new canvases or edit the existing ones whenever they wanted to during the study.

All information generated and used in the CC were assembled and managed in dedicated, secure relational databases inside the KPWA institutional firewall. This database was completely independent of the KPWA Epic EHR system but was populated with weekly extracts from the Epic EHR. The initial prototype website went through two rounds of heuristic testing and iteration with three design experts using the Nielsen’s 10 usability heuristics for interface design [41, 63] and two additional heuristics specific to the goal of the CC tool [43]. The two additional heuristics included the ability of the tool to (1) build a larger picture from complex material and (2) support a process of prioritizing items including identifying and supporting reconciliation of misalignments [43]. The CC website was constructed using a Vanilla JavaScript [69] front end, using D3.js for displaying the nodes and edges. The back-end was constructed using the Django REST framework with data stored securely in a MySQL database. Sockets were integrated into the website to allow simultaneous interaction of participants and interventionists with the same data.

#### 4.2.3 1st Facilitated Conversation.

The interventionist conducted a follow-up telephone interview with the participants to collaboratively engage in the CC to identify connections between values, self-management and health conditions that may be relevant to a specific topic of their care of their choosing (60 to 90 minutes). Participants started by identifying a topic to orient the conversation. Interventionists helped participants choose a conversation topic by suggesting areas of common challenges faced by people with MCC including thinking through a decision, not being able to do something because of health, changes in life, or another topic of a participants’ choice. Participants with the help of the interventionist then selected personal values, self-care tasks and health conditions that may be related to the topic. These were added as bubbles to an open canvas. Interventionists then used motivational interviewing techniques to help participants develop and display the connections between personal values, self-care tasks and health conditions as they related to the participants’ chosen conversation topics. The interventionist took notes throughout the conversation and then created typed summary statements with participants reflecting the connections participants had made from using the CC.

Participants then decided what to share with their provider. They could choose some or all of the following three items from the CC: a list of their values; the diagram of connections between values, self-management tasks and health conditions; and the list of topics potentially relevant for upcoming visits based on the topic of conversation. Family caregivers were asked to add their reflections to the conversation after the participants had provided their perspective. We did not anchor the facilitation conversation to a specific visit since individuals with MCC have multiple visits with many not being planned far in advance. We informed participants and healthcare team members that the conversation and values expressed by the participants are meant to inform their overall care. The day following the facilitated conversation, the interventionist sent a secure email to the participants encouraging them to review the results of the conversation.

#### 4.2.4 Sharing with Healthcare Team and Healthcare Team Visit.

All participants chose to share information from the conversation with their primary care providers. For upcoming visits two or more days away, we shared the results of their facilitated conversation with participants’ providers in the EMR with participants’ consent. We documented what participants selected to share in an encounter note and routed it to the provider with the upcoming visit. Participants were also given the option to contact the study team on their own in advance of healthcare visits to help share the results of their facilitated conversation. Additionally, a reminder of the conversation was sent to providers in an EMR staff message before upcoming appointments (within the next two and a half months).

At the next visit with a primary care team member, we advised providers to acknowledge the CC use and the values being shared. The primary care team member was expected to review shared items from the conversation and potentially use them in care planning where relevant.

#### 4.2.5 2nd Facilitated Conversation.

After a recent visit with one of their study enrolled healthcare team members (their PCP or other enrolled primary care team member (registered nurse, medical assistant, social worker, community resource specialist) or after two and a half months, whichever came first), the interventionist reconnected with individuals with MCC to review and update items shared or engage in a new conversation using the CC. Prior to the 2nd conversation, interventionists sent a secure email asking participants to review the results of their first conversation in the CC. The 2nd facilitated conversation then took approximately 60 to 90 minutes, similar to the 1st facilitated conversation. The interventionist asked participants about their healthcare team visit experience and their reflections on their first use of the CC. The interventionist invited individuals with MCC to consider revising the last conversation which included modifying existing relationships between values, self-management, and health conditions. Alternatively, participants were offered to start a new conversational topic with the CC. If participants had not updated values, self-management tasks, or health conditions, the interventionist helped them reflect their current state at the beginning of the conversation.

At the end of both the first and second conversations, the interventionist asked participants if they had an upcoming appointment with primary care team members and what they may want to share from the CC with them. With the consent of participants, the interventionist communicated the results to the healthcare team members through an encounter note in the Epic EHR.

### 4.3 Evaluation

We conducted post-intervention interviews with individuals with MCC participants and their caregivers remotely by phone after they completed the intervention. Interviews typically lasted 60–75 minutes and followed a semi-structured interview guide to capture perspectives of the individuals with MCC participants on the visit (e.g., how well their providers understand their values, whether their care plan was affected) and their experiences with the CC and the facilitated conversations with the interventionists (e.g., how the first and second conversation differed, how the conversation and/or the visualization tool affected the addressing or sharing of their values). Additionally, we asked the caregivers about how the intervention affected their care and whether they had learned something new about their partners from what their partners had shared with them. We audio recorded the interviews and used a professional transcription service to have the recordings transcribed verbatim.

### 4.4 Analysis

The first author, HR, completed the analysis, with support from two authors, JR and JT, using ATLAS.ti software. The authors analyzed the evaluation interview transcripts using inductive thematic analysis [25] which included open coding, focused coding, and writing up themes that emerged in the process of coding. To begin, the three coders, HR, JR, and JT, coded the same 5 evaluation interview transcripts using an open coding approach to generate and apply provisional codes. The three coders met regularly to compare codes and definitions, refine these into a revised codebook. During focused coding, the first author coded the transcripts using the finalized codebook, met regularly with the other two authors to discuss and clarify emerging themes, reviewed excerpts associated with themes, and wrote theme descriptions to use in this paper. A final set of themes are presented in the findings.

## 5 FINDINGS

In this section, we discuss the five themes generated from our analysis of the post-intervention interview transcripts. Analysis of participant interviews showed that developing visualizations of the connections between personal values, self-management tasks, and health conditions helped participants (1) make sense of the interconnectedness and recognize a road map of the most important issues to manage in their health, (2) feel respected and understood, (3) develop priorities for care conversations with providers, and (4) support changes in behavior aligned with personal values.

### 5.1 Sensemaking of Connections and Core Issues

**Figure 3:**
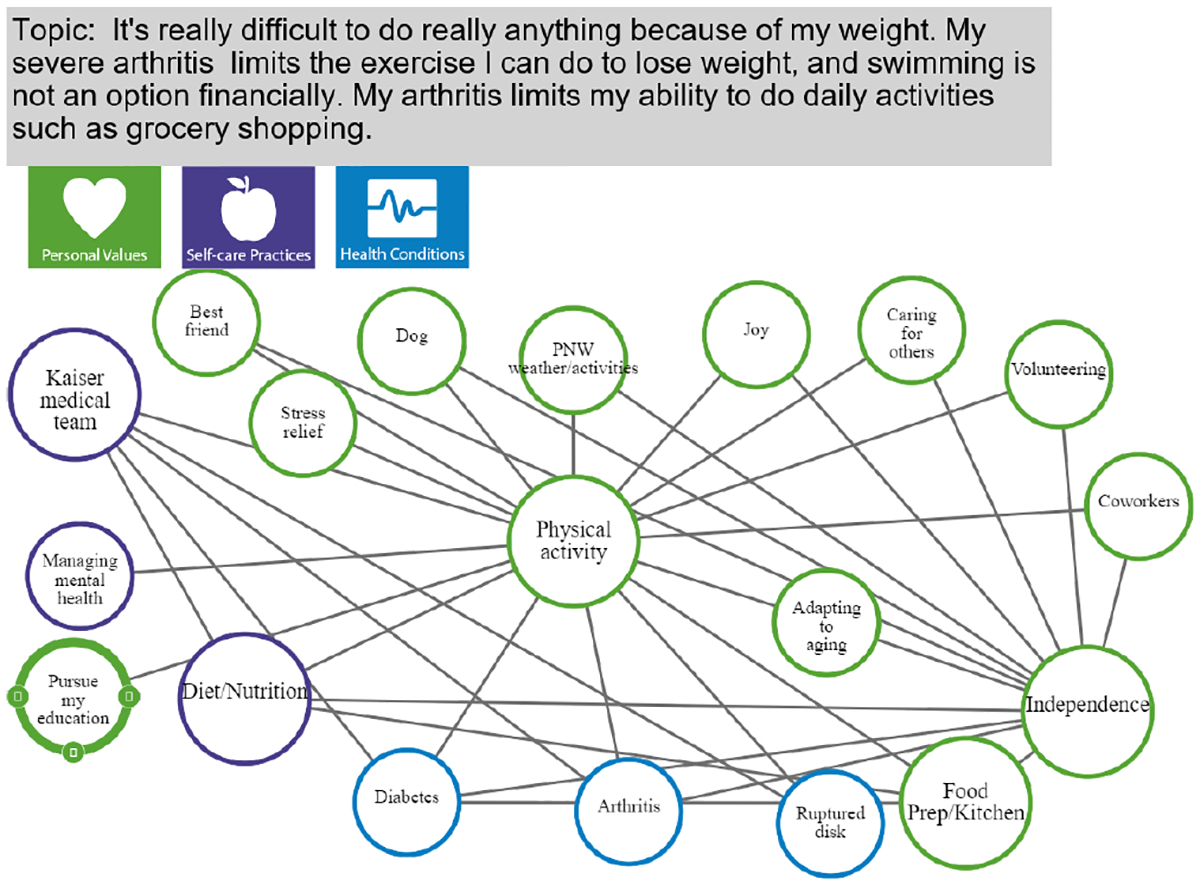
Summary of Facilitated Conversation from Participant. The personal value of physical activity is located in the center as the base issue. It is connected to other personal values (e.g., independence, food prep/kitchen, pursuing education), self-care practices (e.g., managing mental health, diet/nutrition), and health conditions (e.g., diabetes, arthritis, ruptured disk).

The visualized networks rendered from the two sequential conversations facilitated by trained human interventionists played a vital role in sensemaking of the overwhelming number of medical issues that participants had to deal with. The visual organization of the numerous personal values, self-management tasks, and health conditions illustrated on CC made it easier for them to have a roadmap of the issues that they had to deal with and newly revealed their interconnectedness (Figure 3).

I have a laundry list of issues, and it was making my brain get out of control as far as fatality thinking instead of resolving thinking. It gets you out of the pity party a little bit, so you can see where the struggles are. When it’s all on paper, it lines it up like okay, here’s what you need to do. If you stop doing this, this would be better, that kind of thing. But having it all on paper makes it less of a scattered thought process throughout the day.

- P7

One reason visual organization helped with sensemaking was the process helped with externalizing issues. Participants put more significance on a documented summarization of the discussion rather than the discussion itself to validate for themselves the multitude of MCCs they dealt with on a daily basis.

What it did was it took everything – instead of having all these words and thoughts and ideas in my head, it laid them out in front of you on paper. In other words, it’s like a deck of cards. If they’re in your head, it’s a deck of cards. You know all the cards that are in there but when you lay it out on the table to play solitaire, you separate, and you put them in the proper place so you can see the connections.

- P4

Collaborating with trained human interventionists on the visualization also gave participants the opportunity to examine the different parts of their lives and how they connect in ways that they do not get to process daily. One participant (P1) even described the intervention calls as a “soul searching” experience entailing “thinking deeply about certain aspects of [his] life, [his] personality and who [he is].” The intervention calls gave participants dedicated time to pause and reflect while answering the questions asked by the interventionists and visualizing their answers with them.

I understood [how] my new diagnosis of diabetes affects mental sharpness when I don’t eat well - how I need to eat, cook at home. So it really gave me the opportunity to look at specific things and see how they connect in ways I don’t really get to process daily. That was the best part of it.

– P9

The two sequential interventions shared the same structure, but the majority of the participants stated that the visualization helped them realize how a base issue interplayed with their other issues during the first conversation more than during the second conversation. They were not previously aware of this impact that the base issue had on other issues, and they found understanding of its interconnectedness to other issues helped them understand which of the overwhelming set of medical issues they would need to prioritize addressing.

I think the most important part out of that [first] conversation, that exercise, was how interconnected everything was and to be able to see that if this one area was addressed - kind of like a firework that it explodes out from the middle and encompasses the other part.

– P3

Another participant (P12) was experiencing depression and she could not figure out potential contributors. Through the discussion with the interventionists and review of the visualization (the connections between the depression and personal values, health conditions, and self-management tasks), she was able to understand the potential causes of her depression. She found she valued sharing the food that she made with her loved ones, and it gave her joy in life (personal value). However, she could not cook or bake the food that she used to make for her loved ones because of the low-sugar diet (self-management task) that she had adapted for self-managing her diabetes (health condition). She realized her depression was worsening around the holidays because she could not cook or bake the rich and sugary holiday foods she would normally share with her family.

After rendering a network clearly defining the interconnectedness between the personal values and self-management tasks, participants identified and prioritized specific issues to address rather than dealing with all issues at once. Having two sequential conversations provided participants the opportunity to either examine different issues during each conversation and to deepen reflection on the same issue in the second conversation. In the process of dealing with a specific health issue, they examined what personal values, self-management tasks, and other health conditions it was connected to. After examining the interconnectedness, they would think about how they could resolve the health issue and further think about how to improve their communication with their providers. This further connection of sensemaking to the clinical context was unforeseen in prior prototype testing for this study.

I think it was very helpful to me, to be able to look at different issues and each time I did it, I picked a different one. To be able to look at them, and really look at my medical problems and identify what it was, how it was related, and what I can do to make it better, instead of normally just “Oh doctor, I need this, or this is wrong.” To be able to identify what the problem is, what it interacts with, and how to take care of it I think is very important.

– P5

### 5.2 Empowering Individuals with MCC by Respecting and Understanding Their Feelings

Participants stated that they felt more empowered after using the CC with the interventionists. They expressed that they were able to share their feelings, especially concerns and fears, with the interventionists and their providers. Participants indicated that expression of their feelings was more valued in communications with providers during clinical visits since they could show how their feelings were connected to their personal values, self-management tasks, and health conditions. They thought that the CC acted as an acceptable medium to express their feelings with a visualization elucidating the connections between their feelings, their personal values, self-management tasks, and health conditions.

When they [individuals with MCC] talk to their doctor, they don’t say to their doctor, “I’m scared, I don’t like this feeling that I’m having.” **But by using a tool like this, it’s kind of that bridge in between. You can actually verbalize the things that are important in your life, the things you’re concerned about in your life, it meshes it all together there. The important things, the things you want to accomplish. And also you can put your fear in there without being judged, or saying it and sounding like you’re wimping out.** It just makes sense to have something that justifies how you’re feeling and you can look at it and know why you’re feeling it.

– P10

Participants further stated that they felt “lighter, less encumbered, and in touch with themselves (P10)” after sharing their feelings with the interventionists. The trained human interventionists being external to the situation and being non-judgmental helped participants be open and honest about their feelings.

[The first interventionist] allowed me to be totally open and honest about my feelings. I was able to even mention folks by name and I felt pretty good about it, because [the first interventionist] didn’t know them so it allowed me to have a straightforward conversation, because **1) I know that the conversation was anonymous and 2) as I stated, [the first interventionist] doesn’t know the coworkers. So I felt safe in the environment and free to really talk about the experience**, to really get at what I needed to do to just maintain my overall health with the strength that was coming about. I think that’s the gist of it. It was an easy conversation. I felt safe in the environment and the tool once again just helped me put some things into perspective.

– P9

More importantly, the trained interventionists played a pivotal role in making participants feel safe and at ease to share their personal values, self-management tasks, and health conditions. Participants felt actively listened to by the interventionists providing participants as much time as they needed to express themselves and share all that they wanted to share, capturing and summarizing what they had said, and making them feel comfortable.

I felt that both moderators gave me the time to just talk as much as I needed to and share what I wanted to share. I didn’t feel like oh, I got another client, you got to wrap this up. And I’m a talker so I didn’t feel rushed, I really just felt like there was dedicated time for me to think things through, think the tool through.

– P9

The trained interventionists were also valued as guides to help participants dig deeper into what they know and feel. By showing the interconnectedness between participants’ personal values, self-management tasks, and health conditions, interventionists were able to help individuals with MCC gain awareness and understanding of their feelings in relation to the connections they have made together on the CC.

It helped me think about what I really know and feel and that kind of thing. So yeah, I think it helped tie things together very well. It’s easier if somebody walks you through it or you’ve discussed it with it in front of you while talking to somebody, I think that is a better start to being able to use the tool than just going in and seeing the tool come up and maybe a couple examples.

– P5

### 5.3 Perceived Enhancement of Individuals with MCC’s Care

#### 5.3.1 Supporting Individuals with MCC’s Communication about Their Priorities to Providers.

CC helped individuals with MCC identify their priorities which they thought differed from their providers’. One participant (P4) thought his provider’s priority was to resolve immediate issues arising from diabetes (e.g., lowering A1C levels), whereas the participant’s priorities were addressing chronic pain and sleep apnea. CC allowed individuals with MCC to share their priorities with their providers which they felt would increase the providers’ understanding of them and better align their care with what was most important to them. Another participant expressed it this way:

Without this tool [CC], you can go into a doctor and they say your lab test was - whatever. When you’re thinking, “But my foot really hurts.” So you’re on different pages. I think this could be really, really helpful to get both any provider and the patient on the same page, or it could be they don’t get on the same page. That what the doctor thinks is a priority may not ever be my priority, but to be able to identify those is very important.

- P3

Participants also stated that the CC served as “another layer of voice (P10),” allowing them to bring up important issues that they wanted to during the short clinical visit time. CC was an overall agenda to remind the provider of concerns and priorities that their patients wanted to discuss during the visit but normally could not.

The thing the survey does, or the canvas, is to get those things that you don’t have time to talk about normally. I could write them down beforehand - you don’t even think about half the things you want to talk to him about. So again this makes it so he’s got a roadmap to see what I’m concerned about and then he can just look at the canvas and say that was one we wanted to talk about or make notes of it.

– P5

CC also supported participants’ active communication with their providers regarding their priorities during the visits. Participants felt more confident and organized from knowing the whole picture – how their personal values, self-management tasks, and health conditions were interconnected. They stated that they had not previously been able to associate the connections and thus were not able to convey why they were feeling a certain way. With the CC, they were able to communicate their non-physical sides of health (e.g., grief, depression) with an explanation behind their thoughts and feelings.

The communications I learned in your little study helped me verbalize to them what I was feeling so it wasn’t a struggle. It became easier for me to communicate my grief [of losing a loved one] … **I think it added to it - it enhanced the care I was getting.** It brought up certain subjects that normally probably wouldn’t have been addressed in our time limit that we have. … But this helped me keep organized enough to bring up the issues that I was concerned with.

- P10

Most participants were not certain that their providers had reviewed the information they had shared, but they thought that sharing the CC with their providers created a “dedicated moment for them to review” (P9). Although most participants stated that their provider did not explicitly mention that they had reviewed the shared information, participants had experienced a positive change in having a conversation with their providers that was more aligned with their priorities.

The conversation [with my provider] just was different – I don’t know if it’s a result of her reading my thoughts from the study. I don’t know what triggered the relationship with the conversation, but **it was more in line with what I wanted, the approach that I wanted for my health care rather than the cookie cutter**, you have to take these drugs, and if that drug don’t work, you take these drugs. So there had to be more to it than that, because the more drugs she would give me, the less I would want to take them. **So for me to focus on the things that was important in terms of my health, so the conversation went very differently and it allowed me to be more open around my thoughts and feelings about my own health care.**

– P11

#### 5.3.2 Caregiver-Perceived Care Improvement.

Although we had limited caregiver participation, caregivers who engaged thought that they could support their partners better. Caregivers were able to better understand how they could assist their partners. One caregiver expressed it this way:

I guess removing some of his layers made it easier for me, but his mental health work is something that he has to do for himself. So him starting the journey I guess made it easier for me, it allowed for me to be able to assist in conversations.

– CG9

Also, caregivers were able to understand expressions of their partners better and support them in the manner they needed, especially in cases where individuals with MCC were less expressive in explaining why they were feeling a certain way. One participant (P13) had a harder time in expressing how he was feeling, so he would get quiet when he was hurting. His caregiver thought that he was upset at her and would ask him what it was that she did or said that made him upset. This did not help the individual with MCC in resolving the pain and made the caregiver upset which took a toll on their relationship. However, after the interventions, the caregiver learned that the quietness was how her partner expressed experiencing pain and was able to better support him in those times. The caregiver thought that this new understanding had improved their relationship.

Well, prior to these calls when he would get quiet, I would think it was something that I did or said that made him upset. Now since these conversations, I say are you upset with me? “No, I’m just hurting.” Okay, now I understand. I think it’s made our relationship better because I’m not misunderstanding what he’s going through.

– CG13

### 5.4 Supporting Post-Intervention Changes

The discussion with the trained human interventionists and the visualization helped support changes that participants wanted to make. The visualization helped participants realize that they were not dealing with many individual problems, but interconnected problems. This understanding that the problems are related with one another made thinking about changes they needed to make to resolve the problems seem less demanding.

I don’t really think about things that are wrong, I don’t figure out how I’m going to fix it. So it makes it easier to see it on paper, how everything flows together and it’s not seventeen different things making you crazy. It’s not that I’ve figured out how to fix things, it’s a better understanding of how it all works together in order to accomplish being done – fixed.

- P7

As the aim of this study was to understand how to support the development and sharing of connections between personal values and self-management tasks for individuals with MCC, participants’ sensemaking of connections and core issues was expectedly shared by all participants. However, participants’ engagement in changes to their lives was unexpected. Participants who intended to or were making changes after the intervention calls focused on making changes related to the base issue uncovered during the first intervention call. Even though participants came into the intervention knowing what their base issue was, it wasn’t until after realizing its interconnectedness with their other issues that they were more inclined to make changes to alleviate it. For instance, after P12 had found out that her depression was affected by her not being able to make sugary food with her loved ones, she started researching how to make alternative desserts and continued to pursue activities that she valued while keeping to her low-sugar diet to manage her diabetes.

Okay, how can I still do what I want to do? How can I still bake and cook for people to express my love? … so I just decided I’m going to learn to make some keto desserts, I’m going to make it for my family members that are also diabetic. So I did that, I was able to do some research and look at recipes. I found a copycat of the Starbucks Cranberry Bliss bar that was fully keto, I made that. … It was very fulfilling just to see the look on their face when I brought them those desserts and those treats… It was definitely a very great experience for me. **So having that conversation and doing that exercise helped me, like I said, make the best of the situation I had.**

– P12

After the first intervention call, participants were more aware of the impact that actionable behavioral changes could have on their health conditions. This helped them take the next steps to a resolution.

It opened my awareness to the conditions. I wasn’t simply doing things like throwing back the pills, I was actually thinking about why, and with [first interventionist], it brought a new awareness to that. Just talking about it with her helped me go yeah, good, all right. After I talked to her, I actually joined the YMCA…I realized I really had to start moving, so I do aerobics.

- P10

Participants who had started making changes after the first intervention call reflected on those changes during the second intervention call. After participants had implemented a change, they discussed how their personal values were affected by the change and how they could better honor their personal values in pursuing the change.

I started doing things that [the first interventionist] and I had talked about. [The second interventionist] and I - when we talked about it, I could see the benefit of what I had started and I saw the benefit not only for myself but for my clients, of being more open. I saw little things that we tweaked the importance on with [the second interventionist]. **It was like yes, I’m aware - [the first interventionist] and I made the awareness, pointed to this, but I see it, this is really important to me.**

-P10

It is important to note that participants not only reflected on the changes they have made as a result of the intervention calls, but they also continued to reflect on their own. One participant (P2), who had injured her bladder during childbirth and had survived bladder cancer, used the CC as a reminder for her to adapt a new self-management task (regulating her water drinking) to help resolve issues with her bladder.

**I took a couple of screenshots [of the CC] so I have it, and just taking a look at them helps me remember these are [self-management] practices I need to continue**. For instance, I stopped drinking water … the more water I had in my bladder, the more I was leaking and on top of it, it hurts. So I slowed down my water intake, but that’s not helping me. **So when I pulled it up and reviewed page, I was like oh yeah, I’m not drinking enough water, I need to see if that’s going to help with my bladder**.

- P2

### 5.5 Conversation Canvas Limitations/Usage Barriers

Participants perceived a few limitations to CC. First, CC was perceived to be useful by participants only when it was paired with facilitation. Conversation with the interventionists was pivotal.

With [second interventionist] and [first interventionist] is **they ask good follow-up questions, that make me think oh yeah, I got to think about that. So the conversation piece with the canvas is very important.** The canvas on its own? You won’t really get a good response on it because it’s not teaching anything, it’s not a repository holding anything but the canvas image and maybe your personal values, but without an activity to follow along, without it really being a true repository, it’s just a user-friendly tool.

– P9

Participants also said that the CC would be effective in enhancing their care only if the providers review the summary of their priorities. Even though many participants found the reflection exercise valuable, they thought the discussion with the interventionists and the visualization was worthwhile if providers were able to review and apply the acquired knowledge to the patients’ care rather than as a standalone reflection tool with the interventionist.

Although participants faced difficulties in signing into CC because they had lost their sign-in sheet and recovering their user ID and password was time-consuming, they thought that technology experience was a low usage barrier for CC. Participants thought that having the interventionists’ facilitation of using the CC lessened the burden for them and made it particularly easy for them to adapt to using the tool.

So I feel with the moderators who helped me through it, if an aging adult had that type of care, they would be able to facilitate using this particular tool with or without tech experience, because it doesn’t require a lot.

- P9

## 6 DISCUSSION

The aim of the study was to evaluate how the intervention supported the elicitation and sharing of the personal values and priorities of individuals with MCC. In this section, we describe the conjoint and respective roles of the facilitated conversations and CC, the interactive visualization tool, in supporting priorities-aligned care. We elucidate the roles in two aspects: (1) improved communication of priorities through externalization and sensemaking, and (2) improved understanding of multiple care needs and roles in patients’ everyday lives and preparation for change through iterative reflection.

### 6.1 Improved Communication of Priorities Through Externalization and Sensemaking

We found (1) externalization and sensemaking improved communication of priorities, and (2) our tool most closely aligned with the data-frame theory of sensemaking which allows meaningful exploration rather than focusing on actions as the necessary outcome. Our study’s findings contribute to CHI by showing how the intervention addresses design opportunities for improving communication of priorities in chronic illness care suggested by prior work [15, 54].

As with prior studies [15, 54], we have found that externalization helps with the sensemaking of personal values and self-management. Lim et al. challenged the prevalent assumption that patients’ personal values simply require elicitation as patients are already aware of and understand their personal values [54]. They further emphasized the significance of externalizing personal values to bring awareness to and make new discoveries about the interconnectedness of their values, self-care tasks, and health conditions. Both Berry et al. [15] and Lim et al. [54] emphasized the need for trained human interventionists to guide individuals with MCC through the externalizing and sensemaking process.

Compared to prior studies, the potency of sensemaking in this study appeared to be even stronger than what prior development phase studies had discovered [15, 54]. People were making more connections between their values, self-care tasks, and health conditions, and were exploring the connections more deeply. **We associate this new finding with the use of facilitators trained in motivational interviewing, using fully functional interactive visualization and two sequential opportunities for facilitated visualization.**

The conversations with trained human facilitators helped participants clarify information needs that required meaningful exploration. Creating the visualizations with facilitators helped participants overcome difficulties such as not knowing which questions to ask themselves and how to dig deeper into what they know and feel. The visualizations themselves also fortified individuals understanding that they were not dealing with many individual isolated problems, but rather interconnected problems with underlying issues. Our findings of visualization enabling the identification of underlying issues align with prior work by Goyal et al. [35]. Our work and Goyal et al.’s both show that interacting with the visualization made the connections among individual components more apparent and the visualization may have given starting points for the investigation of finding a single underlying potential issue affecting all other issues [35].

The unique contribution of the visualization to the sensemaking of participants echoes findings by Yi et al. [85] on the insight gaining process, specifically “providing overview” and “adjusting”. The interactive visualization uniquely contributed to sensemaking by acting as a base map to refer to in the process of insight gaining of “providing overview” - making sense of and finding which areas needing more investigation, thereby promoting further exploration of the dataset [85]. The facilitated conversation helped individuals with MCCs who did not know where to begin their exploration in the insight gaining process of “adjusting” – exploring a dataset by adjusting the range of selection [85]. By grouping and aggregating information from the large, unorganized dataset (a myriad of personal values, self-management tasks, and health conditions) with trained human interventionists, individuals with MCC’s search and working memory load were greatly reduced, allowing them to use their attention to find higher-level facts and ask new questions [21, 34]. Thus, the trained human interventionist facilitation with the interactive visualization and self-reflecting with interactive visualization respectively enhanced individuals with MCC’s sensemaking process.

**Our findings are most consistent with the data-frame theory of sensemaking** [45, 46]. In the data-frame theory, sensemaking focuses on understanding the mental processes at work while performing functions (e.g., problem detection and identification, forming associations, anticipatory thinking). Meaningful exploration is the goal. Our participants described this type of exploration of the relationships between personal values, self-management tasks, and health conditions [71]. This activity differs from the representation construction model of sensemaking (e.g., Mamykina et al.’s sensemaking-based disease management model [58]), which considers action as a necessary outcome of sensemaking. In the representation construction model, sensemaking is a process of searching for the most optimal external representations of the sought and filtered information to efficiently use them in answering questions related to a specific task [70]. The focus is on creating the external knowledge representations for a specific task [70, 72]. Sensemaking by our participants, in contrast, focused on exploration as the goal without a task or action as a necessary end [46].

Specifically, the first facilitated conversation supported individuals with MCC to perform the above-mentioned functions to define the initial information needs for meaningful exploration. Also, it supported rendering of the initial frame and subsequent iteration of frames composed of personal values, self-management tasks, health conditions, and the interconnections of the three components. The sensemaking process then moves to refining informational needs by elaborating, preserving, or questioning a frame and comparing frames while making further associations between the frame components [45, 46, 71]. We incorporated the process of iteratively refining the initial frames created in the first intervention by employing a second intervention which followed Lim et al.’s suggestion of providing ample opportunities to reshape the individuals with MCC’s information needs continuously [54]. Berry et al. claimed that reflection on values and health in the future allows people with MCC to clarify their healthcare priorities and articulate those with their providers [15]. **Prior to this study, manifestation and refinement of frames through sensemaking in sequential interventions had not been attempted. In our intervention, participants were able to connect the frames to a clinical visit more directly as they felt more confident about their priorities and had the opportunity to think further about how they could better articulate them to their providers.** We acknowledge the intervention would not have succeeded without the participants’ agency to meaningfully explore and communicate with the interventionists. We attribute their active engagement and agency to having the provider visits after the first facilitated conversation and the participants knowing that the results they had selected would be shared with their providers [15].

This study demonstrates the viability of the design opportunities suggested by prior work [15, 54] on improving communication of care priorities. Through sequential interventions with interventionists, patients were able to (1) meaningfully explore their values and their connections to self-management tasks and health conditions and (2) connect sensemaking to a clinical visit more directly.

### 6.2 Improved Understanding of Multiple Care Needs and Roles in Patients’ Everyday Lives and Preparation for Change Through Iterative Reflection

In section 6.1, we elucidated on how the first and second facilitated conversations followed the data-frame theory of sensemaking which focuses on meaningful exploration rather than regarding action as the necessary outcome [45]. However, many participants also unexpectedly talked of changing their behaviors following reflection on the connections between values, self-management work, and health conditions. This was due to the intervention helping individuals with MCC develop a holistic and contextualized understanding of their multiple care needs and roles in their everyday lives and being able to pinpoint the specific issues they wanted to address. Participants who embarked on change also used the second intervention as an opportunity for iterative behavior change and reflection over time. These unexpected findings contribute to CHI by showing how the intervention could reinforce self-management technology design opportunities.

Talk of change often started with deeper reflections on causal or correlational relationships established in the visualization and conversation with the interventionist, especially in the first facilitated conversation where participants uncovered how a base issue interplayed with other connected issues. This finding relates to Saksono et al. [74]’s Experience-Reflection-Insight framework, which illustrates that abstract conceptualization (interpreting the reflected-upon experience) affects dialogic reflection (exploring data relationships and new perspectives), which leads to causal insights. It is also corroborated by Fleck and Fitzpatrick’s taxonomy of five different levels of reflection (R0-R4) [31]. After describing the data laid out in CC without justification or reasons for actions related to the data (R0), participants then examined the relationships between two or more data points (R1). They established casualty or correlation between their previous experiences and their data in dialogic reflection (R2). Then, participants developed a new perspective for reassessing their orientation to perceiving, feeling, or acting in transformative reflection (R3). Participants’ reflections further encompassed critical reflection (R4), which involves taking into consideration aspects that transcend the immediate context, such as socio-cultural contexts and ethical or moral issues. These included participants reflecting on deep-seated beliefs of taking on the burden of managing their health and well-being themselves and internalizing problems they face in self-management tasks. Reflections from R1 to R4 were highly valuable in that they led to frequent and unexpected talk about changing behaviors.

The intervention was meant primarily to support care planning conversations with healthcare providers rather than patients considering and acting on changes in self-management of their health and wellbeing. The unbounding of the reflection process from a planned provider visit or agenda may have enabled reflection that led to considerations for behavior change. In prior development work for CC, Berry et al. emphasized balancing outcome-oriented reflection and exploratory reflection [15]. They stressed that once the outcome was set as visit preparation, pre-existing perceptions of what the patient thinks they could share with their providers were reinforced. However, in this study, rather than making the goal of the interventions to be composing a visit agenda, we asked the patients to help their providers learn about what is important to them (e.g., personal values). This setting supported a reflection process that gave enough room for the participants and the facilitators to ask questions and explore values, feelings, motivations, and behaviors.

Moreover, having a dedicated time and space to externalize their personal values, self-management practices, and health conditions may have made participants more inclined to make behavior changes as it gave them the support with a trained facilitator they needed in knowing what to change and making the changes for themselves [54]. The importance of this type of support in individual behavior modification is consistent with the concept of autonomy support within self-determination theory (SDT), which posits that an “autonomy supportive” environment is essential for establishing care partnerships that lead to patients’ autonomous motivation for healthful self-management through developing individual competence (ability to master environmental challenges skillfully) [73]. Although SDT informed the design of CC, the intent of SDT in the intervention was to change how patients and providers interacted together in care planning, rather than directly influencing an individual’s self-management of their health. Although improving self-management was not the primary aim, facilitated reflection with CC also provided individuals with MCC an opportunity to think about the workload associated with treatment and the impact of the workload on everyday activities and patient identity [28]. New insights from these reflections led naturally to talk of how individuals wanted to change their lives to be consistent with what was most important to them.

Although this study did not primarily aim to improve self-management, our results support the potential of design opportunities in self-management technology in the HCI field [24, 30, 64, 65, 81]. **First, our results support design that incorporates a holistic understanding of the multiple care needs and roles in individuals with MCC’s everyday lives.** Complexity and individuality result from the number of different roles that patients hold in their everyday lives as a person and outside of the clinical setting [4, 64, 65, 81] Our intervention design elicited both personal values and the key types of daily self-management work in those with chronic illnesses including illness, biographical and everyday work [26, 27, 56]. This approach provided a holistic understanding of patients’ everyday lives which has been strongly supported by prior work on self-management technology design in the HCI field [4, 30, 64, 65, 81]. In our study, we found that individuals with MCC having the opportunity to view aspects of their life beyond the clinical context led to an extended understanding of their different roles and responsibilities in their everyday lives. For example, beyond the role of medially managing their own diabetes, P12 had the role of a mother and sister who wanted to bring joy to her family through cooking holiday food. By understanding how each of their various roles and corresponding responsibilities interacted and affected one another, participants were able to prioritize their needs for more sustainable self-management of their overall well-being and health.

**Second, our results also suggest potential for design that supports iterative behavior change and reflection over time in individuals with MCC** [5, 15, 54]. We found that the second facilitated conversation and visualization helped most patients further clarify connections to underlying issues and deepen reflections on changes they are considering in their lives related to their health. Tools for reflection may support keeping track of how individuals with MCC’s perspectives evolve or any discoveries they make about misalignments or desired changes [54]. However, relying on passive automation and predefined presentation of personal data hinders reflective thinking about the past and speculative thinking about the future [5]. Thus, Lim et al. [54] suggested providing ample opportunities to modify after initial elicitation to allow individuals with MCC’s perspectives to evolve. In this study, we conducted two sequential interventions which were not attempted in prior prototype development studies [15, 54]. In the second intervention, participants who had acted upon the changes discussed in the first conversation reflected on their changes. After the second conversation, they further made modifications to their values and/or the connections between their personal values, self-management tasks, and health conditions. Such possibility of iteration seemed to have affected participants’ behavior change execution after defining the base issue affecting all other issues and changes they would need to make during the first intervention. With ample opportunities to engage in mindful and reflective thinking through designing and completing their visualizations, participants were able to further speculate about the future and even modify the changes they made to their lives to be consistent with what was important to them [5]. Thus, although not all participants have executed a considered behavior change, the possibility to examine and iterate on the effects of the changes through a second facilitated visualization proved valuable to participants.

**Lastly, our results demonstrate the potential for designers to use interactive and guided visualization to help individuals managing MCC identify and work through important issues one at a time and in order of priority.** Participants identified and prioritized specific issues to address rather than dealing with all issues at once. Through guided conversations and the interactive visualization tool, participants could understand the problem they chose to focus on, what it interacts with, and how to resolve it. As patients were not previously able to pinpoint what was really making them feel burdened [28] and not aware of their priorities and personal values [54], having the opportunity to identify and choose a specific issue to address one at a time may have decreased the overwhelmingness of MCC care.

## 7 LIMITATIONS

The study had a few limitations. Different interventionists in the first and second facilitated conversations may have impaired participant trust and openness associated with continuity in healthcare relationships. Both facilitators, however, were trained in motivational interviewing, and participants perceived them to be compassionate, easy to talk to, and patient. Participants also explicitly mentioned that the use of two different interventionists did not affect their extent of sharing or opening up to the interventionists. Although using different interventionists did not appear to have adverse effects on reflection, we encourage future studies to empirically investigate the value of using the same interventionist for sequential interventions. We also did not explore how to further support facilitators. Both facilitators were trained and experienced in motivational interviewing and were licensed social workers. Facilitated conversations, however, took longer than originally expected highlighting a need to look more closely at facilitation requirements in future work.

Participants and their partnering providers were recruited from the same integrated healthcare system, and all had health insurance The results of our study may not be generalizable to individuals with MCC in other healthcare systems and settings including those experiencing limited access to health care. The intervention would also be challenging to adopt within the current resources and roles of primary care practices. Although current primary care models often include social workers trained in motivational interviewing, adding this intervention would involve new work and resources that are often not part of traditional primary care. Future work will need to address sustainability and generalizability alongside larger effectiveness studies. Also, the intervention also did not explicitly elicit or incorporate social risks to health including food and housing insecurity and experience of underlying structural inequities like racism, poverty, and environmental health. Although social health factors came up during some intervention conversations, future work should be more comprehensive at including social health factors into the design of planning care priorities.

## 8 CONCLUSION

In this study, we showed how the use of an interactive visualization system in two sequential interventions facilitated by trained interventionists supported patients’ priorities-aligned care. First, it expanded the sensemaking and reflection process of individuals with MCC helping to identify core issues to their health and wellbeing. Second, it made them feel more empowered by respecting and understanding their feelings. Third, it supported priorities-aligned conversations with their providers. We further uncovered the conjoint and respective roles of interactive visualization in the CC tool and the facilitated conversations in support of sharing priorities with healthcare providers. These findings demonstrate the potential for interactive visualization and facilitated conversations to support self-management and care planning aligned with patients’ personal values. While this study focused on improving care planning conversations, our findings on understanding and honoring the personal values of individuals with MCC also supports further use of value-sensitive design in healthcare. Moreover, our findings also reveal opportunities for future work to directly improve self-management. Extending the combination of techniques used in CC could address calls in prior HCI research to support self-management of chronic illness through a holistic lens centered on everyday life [64, 65] that incorporates end-user customization and personalization [65, 68].

## Figures and Tables

**Table 1:** Demographics of Participants with Multiple Chronic Health Conditions

ID	Age	Sex	Race, Ethnicity	Education	Caregiver
P1	79	M	White or Caucasian	> High school	No
P2	61	F	American Indian or Alaska Native and Hispanic and Other	> High school	No
P3	67	F	White or Caucasian	> High school	No
P4	61	M	American Indian or Alaska Native and White or Caucasian	> High school	No
P5	69	M	White or Caucasian	> High school	Yes
P6	70	F	Black or African American	> High school	No
P7	60	F	White or Caucasian	> High school	No
P8	62	F	Black or African American	≤ High school	No
P9	36	M	Black or African American	> High school	Yes
P10	71	F	White or Caucasian	> High school	No
P11	67	F	Black or African American	> High school	No
P12	46	F	Asian	> High school	No
P13	61	M	White or Caucasian	≤ High school	Yes

## CCS CONCEPTS

• Human-centered computing → Empirical studies in HCI; Empirical studies in visualization; Empirical studies in interaction design; • Applied computing → Health informatics.
